# In Situ Study of Twin Boundary Stability in Nanotwinned Copper Pillars under Different Strain Rates

**DOI:** 10.3390/nano13010190

**Published:** 2023-01-01

**Authors:** Shou-Yi Chang, Yi-Chung Huang, Shao-Yi Lin, Chia-Ling Lu, Chih Chen, Ming Dao

**Affiliations:** 1 Department of Materials Science and Engineering, National Tsing Hua University, Hsinchu 30013, Taiwan; 2 Department of Materials Science and Engineering, National Chung Hsing University, Taichung 40227, Taiwan; 3 Department of Materials Science and Engineering, National Yang Ming Chiao Tung University, Hsinchu 30010, Taiwan; 4 Department of Materials Science and Engineering, Massachusetts Institute of Technology, Cambridge, MA 02139, USA

**Keywords:** in situ nanoscopic deformation, nanotwinned copper, twin boundary, detwinning, dislocation activity, atom motion

## Abstract

The nanoscopic deformation of 〈111〉 nanotwinned copper nanopillars under strain rates between 10^−5^/s and 5 × 10^−4^/s was studied by using in situ transmission electron microscopy. The correlation among dislocation activity, twin boundary instability due to incoherent twin boundary migration and corresponding mechanical responses was investigated. Dislocations piled up in the nanotwinned copper, giving rise to significant hardening at relatively high strain rates of 3–5 × 10^−4^/s. Lower strain rates resulted in detwinning and reduced hardening, while corresponding deformation mechanisms are proposed based on experimental results. At low/ultralow strain rates below 6 × 10^−5^/s, dislocation activity almost ceased operating, but the migration of twin boundaries via the 1/4 〈$10\bar{1}$ 〉 kink-like motion of atoms is suggested as the detwinning mechanism. At medium strain rates of 1–2 × 10^−4^/s, detwinning was decelerated likely due to the interfered kink-like motion of atoms by activated partial dislocations, while dislocation climb may alternatively dominate detwinning. These results indicate that, even for the same nanoscale twin boundary spacing, different nanomechanical deformation mechanisms can operate at different strain rates.

## 1. Introduction

Copper with low electrical resistivity has been used as the interconnect in ultra-large-scale integrated circuits for two decades [1]. However, due to the low strength and high diffusivity of Cu, the interconnect structure fails early particularly owing to electromigration or stress migration [2]. Compared to nanocrystalline (nc, with nano grains) materials, nanotwinned materials (nt, with nano-spaced twins in micro grains), such as nt-Cu, are of great interest because of their high strength and improved ductility without scarifying conductivity [3,4]. With twin boundary spacing (TBS) down to the nanometer scale, the nucleation and accumulation of dislocations around twin boundaries (TBs) are responsible for dislocation–retardation strengthening, resulting in the so-called Hall–Petch relationship [5,6,7,8,9,10,11,12,13,14,15,16]. In contrast, when nanosized TBS is reduced below a critical value, TB migration and eventual detwinning, as a consequence of the interaction or penetration (cross-slips) of glissile partial dislocations with/through TBs, causes softening in flow strength, which follows the inverse Hall–Petch relationship [4,8,16]. More recently, the stress-driven motion of kink-like steps and the migration of incoherent TBs (ITBs) was proposed to be one key mechanism of detwinning under a mechanical load as suggested by molecular dynamics simulations [17] and/or under the influences of temperature and electrical current as observed by the electromigration experiments of nt-Cu [18,19].

However, the detailed process of stress-driven ITB migration in nt structures remains unclear, and the strain rate effect on the deformation mechanisms in nanopillars, particularly correlating mechanical responses to the interaction of dislocations with TBs for strengthening or to the ITB migration detwinning for softening using in situ transmission electron microscopy (TEM), has not been pursued. Thus, in this study, the in situ nanoscopic observations of the nanoscale deformation of single-grained, 〈111〉 oriented nt-Cu nanopillars under compression were carried out using in situ and high-resolution TEM. Different strain rates (load control: 2–4 × 10^−5^/s (ultralow), 4–6 × 10^−5^/s (low) and 1–2 × 10^−4^/s (medium); displacement control: 1–2 × 10^−4^/s (medium) and 3–5 × 10^−4^/s (relatively high)) were applied in the in situ TEM experiments to examine the correlation among dislocation activities, stress-driven ITB migration and mechanical responses.

## 2. Materials and Methods

**Figure 1:**
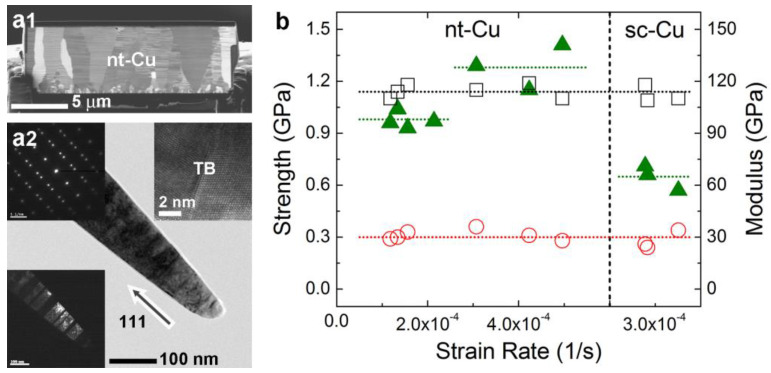
(**a**) FIB-cut thin nt-Cu foil and TEM bright-field/dark-field images, SAD pattern and lattice image of nt-Cu nanopillar; (**b**) elastic moduli (☐), yield strengths (◯) and ultimate strengths (▲) of nt-Cu nanopillars measured at medium to relatively high strain rates of 1–5 × 10^−4^/s, compared to sc-Cu nanopillars measured at relatively high strain rates of 3–4 × 10^−4^/s (dotted lines: average values).

High-purity Cu nanopillar specimens were used for the in situ TEM investigations. A 〈111〉 oriented nt-Cu film with unidirectional nanotwins was deposited on a Ti/Cu-seeded Si wafer by pulse electroplating in a solution containing copper sulfate, hydrogen chloride, and surfactants under high-speed stirring and a current density of 80 mA/cm^2^ [20]. For comparison, single-crystalline Cu (sc-Cu) specimens were also prepared (by rolling and annealing for recrystallization and grain growth to a grain size of 40−200 μm [21]) and investigated. As seen in Figure 1a, thin foils were cut from the specimens along the 〈111〉 direction, following a top Pt protective coating by using a focused ion beam system (FIB, FEI Nova-200, Hillsboro, OR, USA) at a current of 1 nA. From the image in a1, nanocrystalline Cu grains were observed to grow on the Ti/Cu seed layer, and soon the nt-Cu structure with an average TBS of about 35 nm was formed in columnar grains with a size of about 3 μm. The foils were then attached to a C-shape ring and further milled at an ultralow current of 10 pA (to avoid ion bombardment damage) into single-grained, 〈111〉-oriented nanopillars with a tip diameter of below 100 nm and a length of 2–4 μm. From the bright-field and dark-field images of the nanopillar in a2, the nt structure with parallel 〈111〉 TB planes and a TBS of several tens of nanometers was clearly identified. The selected area diffraction (SAD) patterns and lattice images were also examined to verify the structure of the specimens and the longitudinal 〈111〉 direction of the nanopillars.

The in situ TEM compression of Cu nanopillars was performed along the longitudinal direction, by using the PicoIndenter^®^ (Hysitron Inc., Minneapolis, MN, USA) installed in a TEM (JEOL JEM-2100F, Tokyo, Japan). A flat-top indenter with a tip diameter of 1 μm was used to compress the nanopillars in a displacement-controlled mode at a displacement speed of 1 nm/s to yield relatively high strain rates of 3–5 × 10^−4^/s and at a speed of ≤0.5 nm/s to yield medium strain rates of 1–2 × 10^−4^/s. In addition, the compression was carried out in a load-controlled mode, by holding loads from 5 down to 1 μN, to yield medium strain rates of 1–2 × 10^−4^/s, low strain rates of 4–6 × 10^−5^/s and ultralow strain rates of 2–4 × 10^−5^/s, respectively. The load-versus-time curves were provided by the machine, and the displacements of the indenter tip at different stages were measured from the recorded in situ images. As the original shape of the nanopillar tips is axisymmetric and slightly tapered, the average diameters of the nanopillars around the pillar tips were used to calculate the pillar-to-indenter contact areas; the diameters that increased during compression at different stages were measured from the captured in situ images to calculate the instant contact areas. The instantaneous stresses at the pillar tips were calculated from the applied load divided by the instantly measured cross-sectional area, and the approximate strains of the pillars were calculated from the indenter displacement divided by the pillar length to obtain the near-true stress-to-strain responses of the nanopillars.

## 3. Results and Discussion

### 3.1. Stress–Strain Responses of Cu Nanopillars

**Figure 2:**
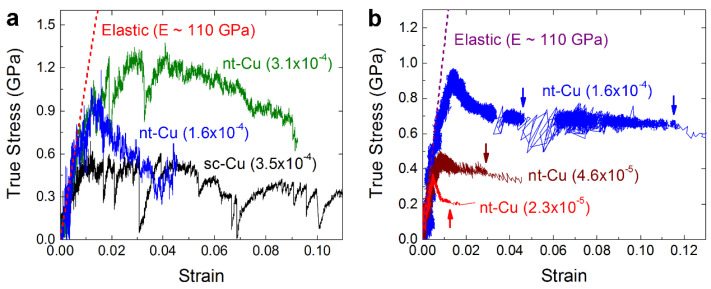
Typical near-true stress–strain curves of in situ TEM compression of nt-Cu nanopillars: (**a**) at relatively high to medium strain rates (displacement-controlled, compared to sc-Cu nanopillar); (**b**) at medium to ultralow strain rates (load-controlled; arrows: detwinning and rapid plastic deformation).

The in situ nanoscopic analyses of single-grained nt-Cu nanopillars show the effect of strain rate on the activation of dislocations and TB migration. As seen in the corresponding mechanical properties and near-true stress–strain curves of compression measured at different strain rates in Figure 1b and Figure 2, respectively, all the nanopillars deformed elastically at the beginning stage with a modulus of about 110 GPa and yielded at a stress of about 0.3 GPa. The modulus is slightly lower than the typical value of Cu, about 120–140 GPa [21], especially in the stiff 〈111〉 direction, possibly due to an underestimation of stress and overestimation of strain during the compression of nanopillars, particularly with a slightly tapered conical tip. The yield stress of the single-grained nt-Cu nanopillars measured in the present study is the same as that of sc-Cu nanopillars but somewhat lower than the reported value of bulk polycrystalline nt-Cu with a similar TBS, about 0.7 GPa [5,6,9], because this in situ study nanoscopically detects the very early nucleation of dislocations and the onset of plastic deformation. For the sc-Cu nanopillars having little substructural influences, dislocation activities simply dominated their plastic deformation: the accumulation or clustering of dislocations induced work hardening to an ultimate stress around 0.65 GPa, and the long-range motion or sudden bursts of dislocations towards the surface, and the subsequent dislocation starvation [22,23,24] then caused the observed large stress drops and considerable stress fluctuations.

In comparison, the nt-Cu nanopillars that were deformed at similar, relatively high strain rates (3–5 × 10^−4^/s) exhibited a much stronger work-hardening capacity than the sc-Cu nanopillars, which could be attributed to the intense interaction of dislocations with TBs as observed below. The ultimate strength of the nt-Cu nanopillars, about 1.28 GPa, exceeds that of bulk polycrystalline nt-Cu, 0.8 GPa at a TBS of 35 nm with a similar strain rate around 6 × 10^−4^/s [7,8,11], probably owing to the TBs completely perpendicular to the loading direction in the present study where dislocation activities were confined in a small volume. However, as seen in Figure 1b and Figure 2, a reduction in strain rate from the relatively high range of 3−5 × 10^−4^/s to the medium range of 1–2 × 10^−4^/s and the low range of below 6 × 10^−5^/s resulted in a decrease in ultimate strength from 1.28 to 0.98 GPa and an even lower value, which is discussed below in detail.

### 3.2. Deformation Behavior at Relatively High Strain Rates

**Figure 3:**
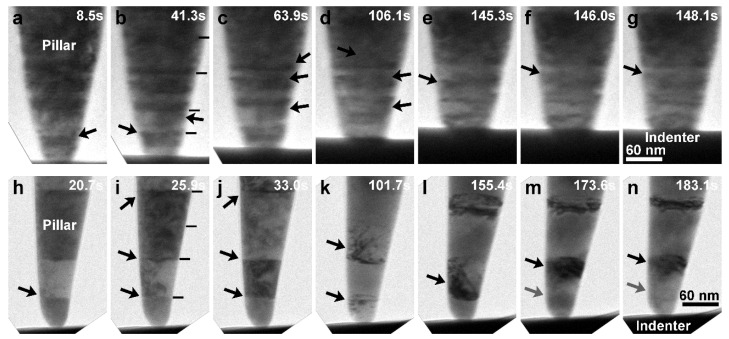
In situ TEM observations (images rotated) of nanoscopic deformation of nt-Cu nanopillars: (**a**–**g**) under compression at a relatively high strain rate of about 3.1 × 10^−4^/s; (**h**–**n**) under compression at a medium strain rate of about 1.3 × 10^−4^/s (short bars: TBs, black arrows: dislocation activities, grey arrows: detwinning).

From the in situ TEM observations of nanoscopic deformation of nt-Cu nanopillars in Figure 3a–g and Supplementary Video S1, the strong work-hardening capacity at relatively high strain rates (3–5 × 10^−4^/s) was attributed to the intense interaction of dislocations with TBs as expected. Upon yielding, dislocations nucleated at the pillar tip and at the first TB above the tip (Figure 3a) and soon were distributed at several TBs (Figure 3b,c) due to cross-slips at the TBs [7,9,11,12,13,15,16,17]. Compared to the long-range dislocation activities in sc-Cu nanopillars, relatively uniform accumulation and clustering of dislocations at the first several TBs (Figure 3d) were observed. As the dislocation clusters were emitted from the TBs and glided towards the next TBs (Figure 3e,f), their motions were mostly confined within twin grains [6,7,11,15], rather than with large extensions to the surface of the pillar, therefore causing stronger hardening than the sc-Cu nanopillars. The pileups at the TBs (Figure 3g) led to an ultimate stress as high as 1.28 GPa. Afterwards, some short-range dislocation activities caused the stress to slightly decrease.

### 3.3. Deformation Behavior at Medium to Low Strain Rates

**Figure 4:**
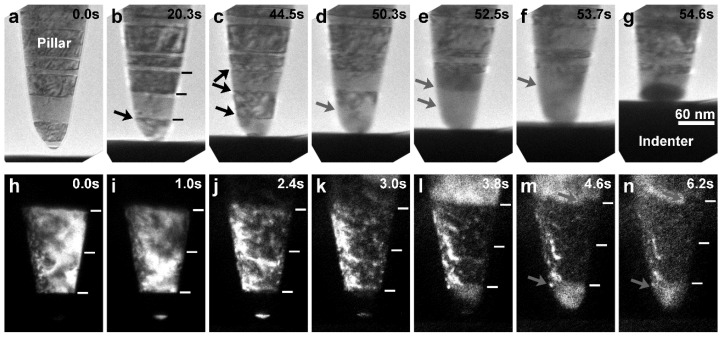
In situ TEM observations (images rotated) of nanoscopic deformation of nt-Cu nanopillars: (**a**–**g**) under compression at an ultralow strain rate of about 2.3 × 10^−5^/s (short bars: TBs, black arrows: dislocation activities, grey arrows: detwinning); (**h**–**n**) under compression at an ultralow strain rate of about 2.0 × 10^−5^/s (dark-field images; short bars: TBs; grey arrows: TB roughening step).

As revealed in Figure 3h–n above (and Supplementary Video S2) and Figure 4 below (and Supplementary Video S3), the decrease of ultimate strength for the nt-Cu nanopillars deformed at a reduced strain rate from the relatively high range (3–5 × 10^−4^/s) to the medium range (1–2 × 10^−4^/s) and low range (<6 × 10^−5^/s) was owing to an altered deformation mechanism: TB migration (detwinning). At medium strain rates as noticed in Figure 3h–n, dislocations similarly nucleated first at the pillar tip. Then, the first TB yielded (Figure 3h), and dislocations uniformly distributed and clustered at several TBs (Figure 3i,j). The accumulation and confined motion of dislocation clusters were observed to cause strain hardening (Figure 3k). However, as the dislocation clusters glided towards the next TBs continually (Figure 3l,m), the detwinning of the first pair of twin grains (Figure 3m,n) was observed (as also seen in the original, captured in situ TEM images at a higher magnification in Supplementary Figure S1a,b). When detwinning occurred, strain hardening ceased at 0.98 GPa, and the stress began to decrease to a lower level (Figure 2a).

The detwinning aspect can be looked into in more detail together with Figure 4 (and Supplementary Video S3), under a load-controlled compression mode. At an ultralow strain rate of 2.3 × 10^−5^/s, soon after the nucleation of dislocations at the first TB (Figure 4a) and the very few initial accumulation around several TBs (Figure 4b,c), the first TB started detwinning (Figure 4d,e; as also seen in the original, captured in situ TEM images at a higher magnification in Supplementary Figure S1c,d), and the detwinning of the second TB occurred successively (Figure 4e,f), resulting in the transformation of the strong nt structure to a weaker sc structure and consequently the rapid plastic deformation of the pillar tip (Figure 4g). The in situ dark-field observation of the nt-Cu nanopillar under compression at an ultralow strain rate of 2 × 10^−5^/s in Figure 4h–n additionally indicates the roughening of TBs at the very early stage of compression (only in a few seconds) when the detwinning started.

**Figure 5:**
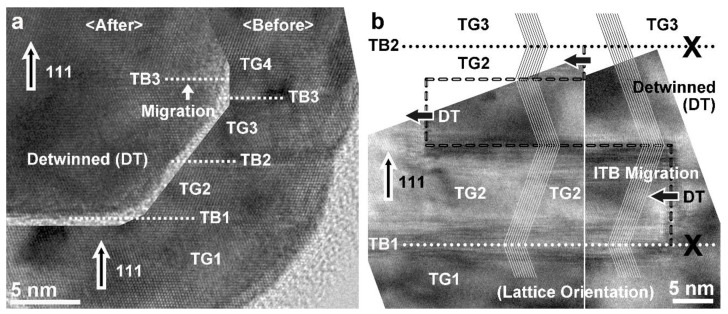
High-resolution TEM lattice images of nt-Cu nanopillars: (**a**) at pillar tip, before and after in situ TEM compression (test interrupted at a slight strain) at a low strain rate of about 5.3 × 10^−5^/s, showing {111} TB migration (TB3) and detwinning (TG2 and TG3); (**b**) several twin grains away from pillar tip, after in situ TEM compression (test interrupted at a slight strain) at a low strain rate of about 4.5 × 10^−5^/s, showing {$1\bar{2}1$} ITB migration (thick arrows) and detwinning (the right part of TG2) (TG: twin grain, DT: detwinned; dotted lines: original TBs, “X”: eliminated original TB sections, dashed lines: newly formed TBs and ITBs).

The high-resolution TEM lattice images of nt-Cu nanopillars before and after compression (test interrupted at a slight strain) at low strain rates of 5.3 × 10^−5^/s and 4.5 × 10^−5^/s, as provided in Figure 5 (and also the original high-resolution TEM lattice images in Supplementary Figure S2), further verify the twin plane migration and detwinning. At the pillar tip in Figure 5a, TB3 migrated upwards for a distance of several atomic layers, TB2 was eliminated, and the two twin grains (TG2 and TG3) between TB1 and TB3 merged into one twin grain. In the other pillar, at a distance of several twin grains away from the tip as shown in Figure 5b, several ITBs were newly formed and migrated (as indicated by the thick arrows) towards the left side of the pillar for different distances, then causing the stepped detwinning of the right part of the original twin grain (TG2) and also the elimination of the right sections of TB1 and TB2.

### 3.4. Detwinning Mechanisms at Different Strain Rates

**Figure 6:**
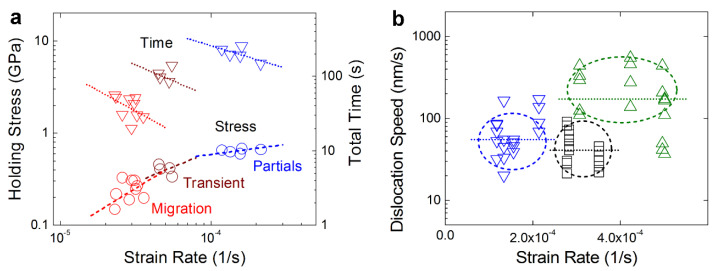
(**a**) Holding stress (◯) and total time to detwinning (▽) versus strain rate plots for in situ TEM compression (load-controlled) of nt-Cu nanopillars (blue: medium strain rates of 1–2 × 10^−4^/s, brown: low strain rates of 4–6 × 10^−5^/s, red: ultralow strain rates of 2–4 × 10^−5^/s); (**b**) dislocation gliding speeds during in situ TEM compression (displacement-controlled) of nt-Cu nanopillars (△: relatively high strain rates of 3–5 × 10^−4^/s, ▽: medium strain rates of 1–2 × 10^−4^/s) and sc-Cu nanopillars (☐: relatively high strain rates of 3–4 × 10^−4^/s).

The detwinning is reflected on the lower stresses in the holding stress-versus-strain rate plots of medium and low strain rate compression during load holdings in Figure 6a. Under relatively high strain rates, as observed above in Figure 3a–g, intense dislocation activities (such as rapid dislocation accumulation at TBs to form a high Peierls stress barrier) normally resulted in the observed strengthening. However, as seen in Figure 3h–n and Figure 4, reduced strain rates induced the detwinning softening of nt-Cu nanopillars, for which the strain rate-governed dislocation gliding speeds are provided in Figure 6b (measured from the in situ images) could be responsible. In the sc-Cu nanopillars deformed at relatively high strain rates, the dislocation gliding speed ($\bar{v}$ ~ 41 nm/s) and the dislocation density (ρ ~ 10^−4^/nm^2^) accorded with the Orowan equation $\dot{\varepsilon }=1/2\rho b\bar{v}$ (b: {111} Burgers vector ~ 0.21 nm) [25]. In the nt-Cu nanopillars at relatively high strain rates, both the dislocation gliding speed ($\bar{v}$ ~ 172 nm/s) and dislocation density (ρ ~ 6.8 × 10^−4^/nm^2^) were higher than those in the sc-Cu nanopillars, suggesting a higher capacity of work-hardening as a result of aforementioned intensive dislocation pileups at TBs [6,7,8,9,11,14,15].

**Figure 7:**
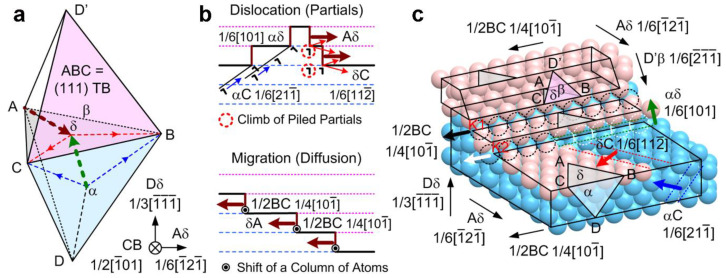
(**a**) Thompson tetrahedron (ABC {111} TB, Dδ 1/3 〈 $\bar{1}\bar{1}\bar{1}$〉 compression direction, CB 1/2 〈$\bar{1}01$〉 viewing direction). (**b**) Detwinning mechanisms by the δA〈$1\bar{2}1$〉 migration (brown arrows) of {$1\bar{2}1$} ITBs (brown lines): (top) at medium strain rates, via the climb or cross-slips of partial dislocations (incident αC 1/6 〈$21\bar{1}$〉 partial → δC 1/6 〈$11\bar{2}$〉 glissile + αδ 1/6 〈101〉 stair-rod); (bottom) at low/ultralow strain rates, via stress-driven 1/2BC 1/4 〈$10\bar{1}$〉 kink-like motion of atoms. (**c**) Schematic models of 1/2BC 1/4 〈$10\bar{1}$〉 kink-like motion and the interaction of 1/2BC 1/4 〈$10\bar{1}$〉 kink with αC 1/6 〈$21\bar{1}$〉 partial at low/ultralow strain rates (blue atoms: below TB, pink atoms: above TB). The 1/2BC 1/4 〈$10\bar{1}$〉 shift (black arrow) of a row of blue atoms (atom by atom, black-dash-circled) at kink K1 align the row of blue atoms with the pink atoms and hence detwin the row (the lattice below TB). At medium strain rates, when an activated αC 1/6 〈$21\bar{1}$〉 partial (blue dashed lines and blue arrow) is incident to and interact with a 1/2BC 1/4 〈$10\bar{1}$〉 kink, a row of vacancies (atom by atom, white-dash-circled) is left at kink K2 (1/2BC 1/4 〈$10\bar{1}$〉 + incident αC 1/6 〈$21\bar{1}$〉 → a row of vacancies + αδ 1/6 〈101〉 stair-rod (green dashed lines and green arrow) + δC 1/6 〈$11\bar{2}$〉 glissile (red dashed lines and red arrow)). The 1/2BC 1/4 〈$10\bar{1}$〉 kink-like shift ceases operating (white arrow), and the δC 1/6 〈$11\bar{2}$〉 glissile alternatively dominates detwinning.

However, the slower dislocation gliding ($\bar{v}$ ~ 55 nm/s) at medium strain rates would reduce dislocation pileups and intersections but would provide more sufficient time for triggering the dissociation of 1/6 〈$21\bar{1}$〉 partial dislocations into 1/6 〈$11\bar{2}$〉 glissile partials [26,27,28,29] or for the cross-slips of partials among twin grains [30], likely causing detwinning via the “partials” detwinning mechanism illustrated in Figure 7a,b (top). Consistently, as seen in the right region of Figure 6a among the three strain rate groups, the holding stress-versus-strain rate plot fits approximately the relationship of $\dot{\varepsilon }=10^{-3}\times \sigma^{4.5}$, matching a “dislocation climb” mode of creep, and the response time-versus-strain rate plot also appears to follow the conventional creep-like relationship of $t=c\times {\dot{\varepsilon }}^{-d}$ (herein *d* ~ 0.6) where the response time is inversely proportional to the strain rate [25].

When the strain rate decreased to a low or even ultralow level, under which the activities of partial dislocations almost ceased operating at a compressive stress of about 0.25 GPa as observed in the experiments, surprisingly, the detwinning did not decelerate but instead occurred much more rapidly compared with the case of medium strain rates as revealed by the short response time presented in the left region of Figure 6a. Different holding stress-versus-strain rate relations are found in these regimes: $\dot{\varepsilon }=10^{-3.7}\times \sigma^{1.5}$ at low strain rates (the “transient” region) and $\dot{\varepsilon }=10^{-3.9}\times \sigma^{1.0}$ at ultralow strain rates (the “migration” region), being likely a “diffusion flow” mode of creep. In both regimes, the response time-versus-strain rate plots also follow the creep-like relationship that $t=c\times {\dot{\varepsilon }}^{-d}$ (herein *d* ~ 0.9 and 1.1). This type of relationship is similar to that for grain boundary sliding, which induces the softening of nc materials [31,32]; however, no strain-rate dependent softening mechanism has been reported yet for nt pillars [33]. This finding suggests that an alternative mechanism, i.e., the “diffusion flow” mode (stress-driven motion of atoms) instead of the “dislocation climb” mode (climb or cross-slip of partials) [18,19], dominates the rapid detwinning softening of the nt-Cu at low/ultralow strain rates.

### 3.5. Detwinning by Kink-like Motion of Atoms

For a Σ3 〈110〉 {111} nt-Cu structure, {$1\bar{2}1$} ITB steps that originated at a free surface or defective {111} TB [17] are proposed to migrate via kink-like motion (the shift of a row of atoms) by 1/4 〈$10\bar{1}$〉, as driven by thermal energy, electron force or mechanical stress [17,18,19]. The ITB migration speed is proposed as $v=MP=M_{0}exp\left(-Q/kT\right)\left(2\gamma /TBS\right)$ (M: mobility of ITBs, $M_{0}\approx 5.6\times 10^{12}-1.3\times 10^{13}$, P: driving force to ITBs, Q: activation energy ~ 0.9 eV, k: Boltzmann constant, T: temperature, γ: {111} TB energy ~ 24 mJ/m^2^) [18]. For nt-Cu with TBS of 35 nm at 300 K, without external force (intrinsic P ~ 1.37 MPa, calculated as referred to [19]), the ITB migration speed is estimated to be as low as 0.006–0.014 nm/s. In the present study of nanopillar compression, as illustrated in Figure 7b,c (bottom), the kink-like motion by $\vec{b}=$ 1/4 〈$10\bar{1}$〉 is believed to cause the successive migration of the newly formed {$1\bar{2}1$} ITBs for detwinning as observed in Figure 5b. Under a compressive stress of P ~ 0.25 GPa, the equivalent radial tensile stress [25] will facilitate the 1/4 〈$10\bar{1}$〉 kink-like motion, and the ITB migration speed is estimated to be as high as 1.1–2.6 nm/s. That is, the ITB migration distance approaches 55–130 nm in 50 s, which agrees well with the above experimental observations.

It reflects a fact that, without significant dislocation activities at low/ultralow strain rates, kink-like motion-induced detwinning can occur even more rapidly than dislocation climb-caused detwinning (herein and in literature, ex. 0.3–1.4 nm/s at 0.45 GPa for TBS of 5–15 nm [34]) because, at the nanoscale, a gradient stress may drive the fast diffusion flow of atoms at the surface or interface at a low strain rate before the nucleation of dislocations [35,36,37,38]. As presented in Figure 7c (and also Supplementary Figure S3, top layers of atoms removed for clearer illustrations), the kink is the step lying at the intersection of the gliding plane and the TB plane, and the kink-like motion of atoms denotes the stepped shift of atoms along the same vector as that of the kink, 1/4 〈$10\bar{1}$〉 [18]. The shift of a row of atoms at the kink (atom by atom, ex. the movement of atoms at the bottom sites of the orange hexagon in Figure S3) will hence detwin the row of atoms below the TB. However, as the strain rate increases to the medium range, dislocations are activated; typical 1/6 〈$21\bar{1}$〉 partials interact with the 1/4 〈$10\bar{1}$〉 kinks and interfere with the motion of the kinks so the diffusion of atoms is “frozen” prior to the onset of displacive plasticity [35,36]. The kink-like motion-induced detwinning is accordingly suppressed. As illustrated in Figure 7c (and Figure S3), the 1/6 〈$21\bar{1}$〉 partials are dissociated into 1/6 〈$11\bar{2}$〉 glissile partials and 1/6 〈101〉 stair-rods. The interaction of the row of atoms at the kinks with the 1/6 〈101〉 stair-rods will leave a row of vacancies. Thereafter, the fast 1/4 〈$10\bar{1}$〉 kink-like motion ceases proceeding, but only the slow climb of the 1/6 〈$11\bar{2}$〉 glissile partials remain interacting with TBs, so the overall detwinning process is decelerated.

**Figure 8:**
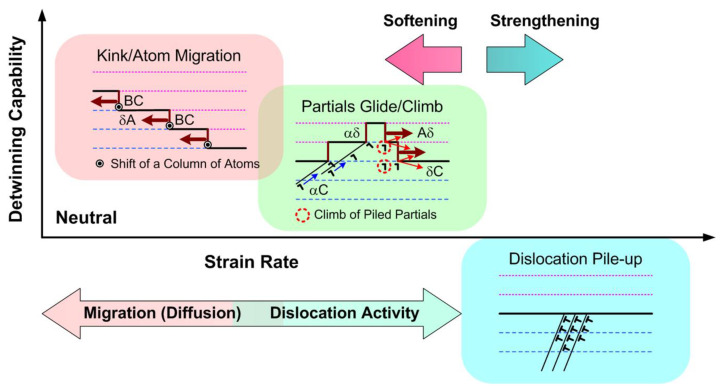
Strengthening/softening mechanisms that operate in nt structures at different strain rates: (bottom right) dislocation pileups for strengthening at relatively high strain rates, (center) partial dislocation climb mode of detwinning at medium strain rates, and (up left) kink-like motion mode of detwinning at low/ultralow strain rates.

The above observations suggest the possibility that, even for the same TBS, different nanomechanical mechanisms can operate at different strain rates, as illustrated in Figure 8: (1) dislocation pileups for strengthening at relatively high strain rates, (2) partial dislocation climb mode of detwinning at medium strain rates, (3) the stress-driven ITB migration via the kink-like stepped motion of atoms possibly to induce detwinning at low/ultralow strain rates. These findings point out an important issue to be carefully investigated: a low strain rate may result in rapid detwinning of nt-Cu and possibly catastrophic failure of nanoscale Cu interconnects even under relatively small residual or thermal stresses.

## 4. Concluding Remarks

The nanoscale structural stability-to-mechanical response correlations of nt-Cu nanopillars were investigated using in situ nanoscopic observations of deformation at different strain rates. Consistent with experimental results, we propose the following nanomechanical deformation mechanisms depending on strain rates. For the same TBS, either dislocation pileups or ITB migration determines nt-Cu strengthening or softening, depending on the strain-rate range. When the strain rate is at a relatively high range of 3–5 × 10^−4^/s, dislocation gliding and pileups are activated for strengthening. When the strain rate is below 6 × 10^−5^/s, dislocation activity almost stops operating, but the ITB migration via the fast 1/4 〈$10\bar{1}$〉 kink-like motion (diffusion flow) of atoms governs rapid detwinning. For the strain rate in-between, the detwinning speed is reduced due to the interfered kink-like motion by partial dislocations, while dislocation climb alternatively dominates detwinning.

## Data Availability

Not applicable.

## Supplementary Materials

The following supporting information can be downloaded at: https://www.mdpi.com/article/10.3390/nano13010190/s1, Figure S1: Captured in situ TEM images of nanoscopic deformation of nt-Cu nanopillars; Figure S2: Original high-resolution TEM lattice images of nt-Cu nanopillars; Figure S3: Schematic models of the kink-like motion of atoms and the interaction of kink with partial dislocation (top layers of atoms removed). Video S1: In situ TEM observation of nanoscopic compression of nt-Cu nanopillar at a high strain rate of about 3.1 × 10^−4^/s (10 times speed up); Video S2: In situ TEM observation at a medium strain rate of about 1.3 × 10^−4^/s (10 times speed up); Video S3: In situ TEM observation at low to ultralow strain rates of about 2.3 × 10^−5^/s (5 times speed up).
