# Supplementary material for: In Situ Study of Twin Boundary Stability in Nanotwinned Copper Pillars under Different Strain Rates
Source: Nanomaterials (Basel). 2023 Jan 1;13(1):190. doi: 10.3390/nano13010190 (PMC9823832; doi:10.3390/nano13010190)
Supplement: Supplementary file 1 [file nanomaterials-13-00190-s001.zip › nanomaterials-2127530-supplementary-Figures.pdf]

Supporting Information

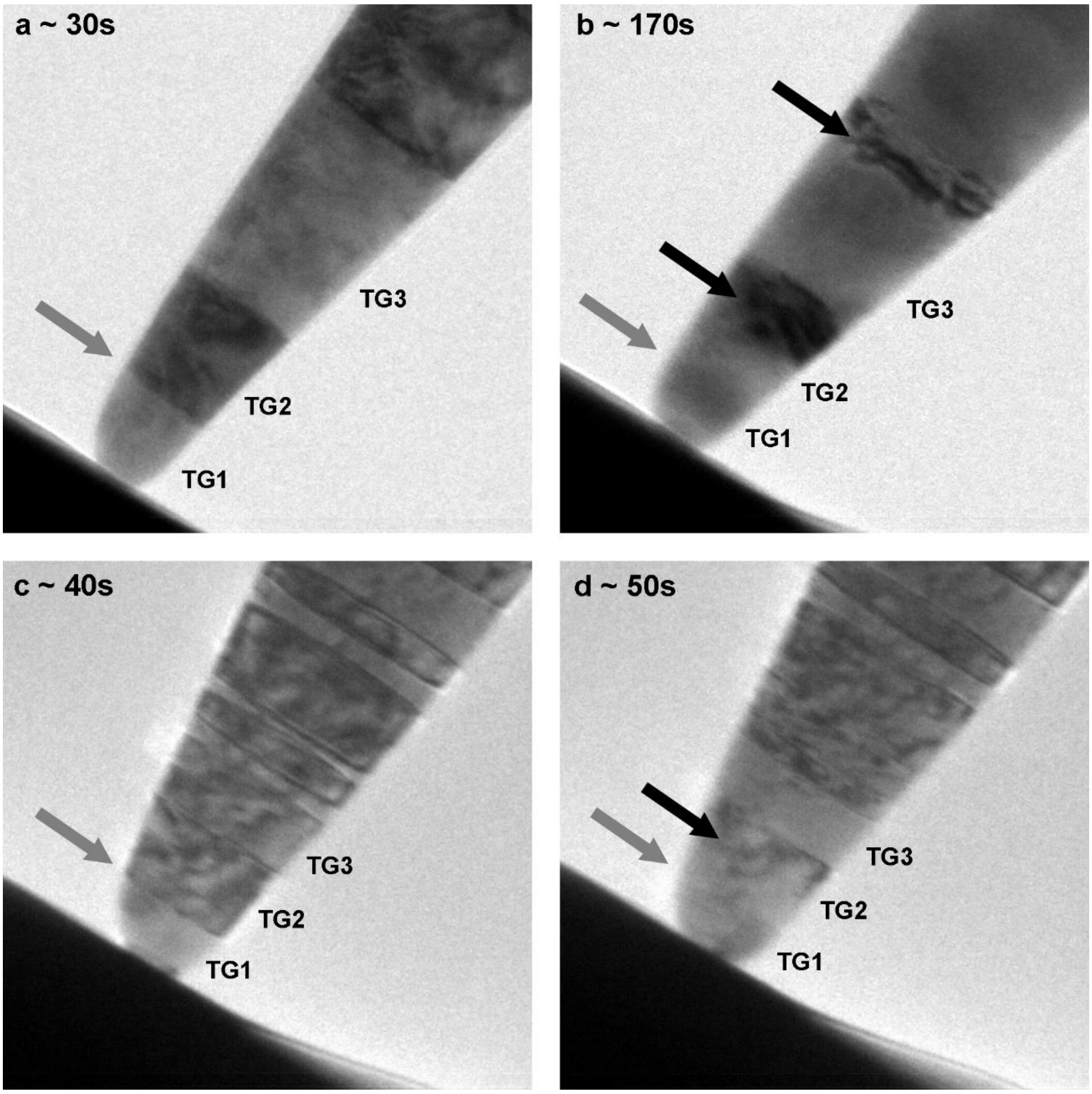

Figure S1. Captured *in-situ* TEM images of nanoscopic deformation of nt-Cu nanopillars: under compression at a medium strain rate of about 1.3×10^{-4}/s for different time: (a) about 30 s, (b) about 170 s (black arrows: dislocation activities, grey arrows: detwinning); under compression at an ultralow strain rate of about 2.3×10^{-5}/s for different time: (c) about 40 s, (d) about 50 s (black arrows: dislocation activities, grey arrows: detwinning).

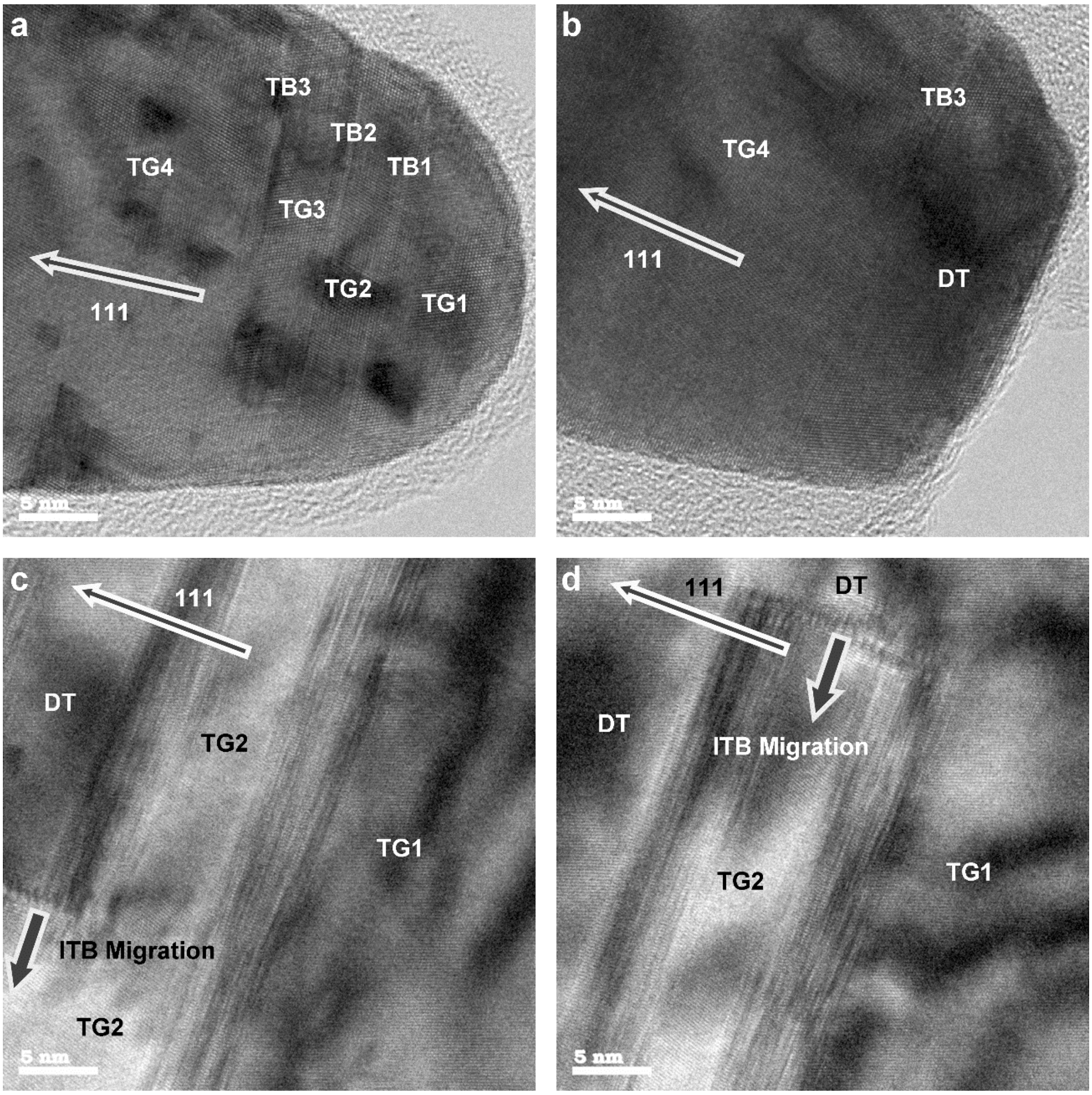

Figure S2. Original high-resolution TEM lattice images of nt-Cu nanopillars: at the pillar tip, (a) before and (b) after *in-situ* TEM compression (test interrupted at a slight strain) at a low strain rate of about 5.3×10^{-5}/s /s, showing {111} TB migration (TB3, along the ⟨111⟩ direction) and detwinning (TG2 and TG3); several twin grains away from the pillar tip ((c) the left part and (d) the right part of the pillar sample), after *in-situ* TEM compression (test interrupted at a slight strain) at a low strain rate of about 4.5×10^{-5}/s, showing {1–21} ITB migration (along the thick arrows) and detwinning (the right part of TG2; TG: twin grain, DT: detwinned).

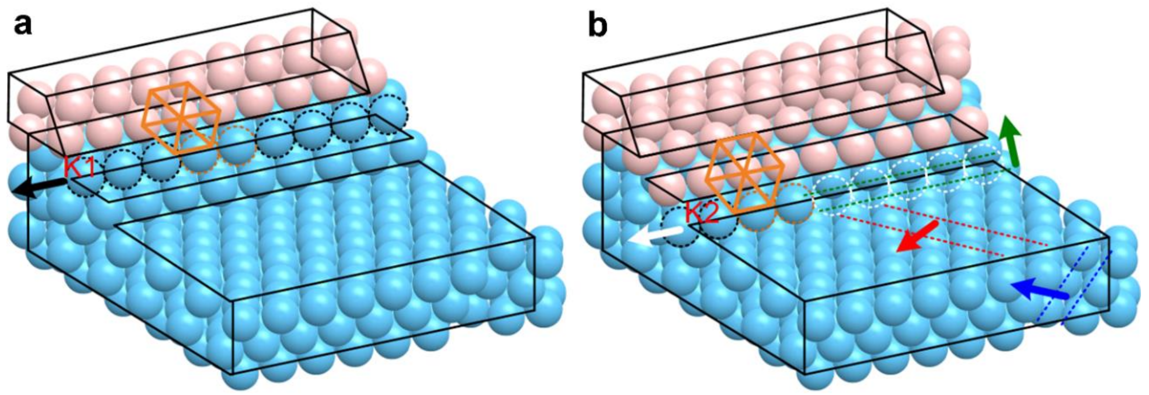

Figure S3. Schematic models of the kink-like motion of atoms and the interaction of kink with partial dislocation (referred to Figure 7c, top layers of atoms removed for clearer illustrations; blue atoms: below TB, pink atoms: above TB). (a) The 1/4 ⟨10–1⟩ shift (black arrow) of a row of black-dash-circled blue atoms at kink K1 (atom by atom, *ex.* the movement of two orange-dash-circled blue atoms to the bottom-left and bottom-right sites of the orange hexagon) will align the row of blue atoms with the pink atoms to extend the close-packed {111} plane above TB and hence detwin the row of atoms (the lattice below TB). (b) When an activated 1/6 ⟨21–1⟩ partial dislocation (blue dashed lines and blue arrow) is incident to and interact with a 1/4 ⟨10–1⟩ kink, the 1/6 ⟨21–1⟩ partial dislocation will be dissociated into a 1/6 ⟨101⟩ stair-rod (green dashed lines and green arrow) and a 1/6 ⟨11–2⟩ glissile (red dashed lines and red arrow). The row of the dash-circled blue atom sites will be emptied by the 1/6 ⟨101⟩ stair-rod to form a row of white-dash-circled vacancies at kink K2 (atom by atom, white-dash-circled). The 1/4 ⟨10–1⟩ kink-like shift will cease operating (white arrow), and the 1/6 ⟨11–2⟩ glissile will alternatively dominate the detwinning process.
