# Supplementary material for: In Situ Study of Twin Boundary Stability in Nanotwinned Copper Pillars under Different Strain Rates
Source: Nanomaterials (Basel). 2023 Jan 1;13(1):190. doi: 10.3390/nano13010190 (PMC9823832; doi:10.3390/nano13010190)
Supplement: Supplementary file 1 [file nanomaterials-13-00190-s001.zip › nanomaterials-2127530-supplementary-Videos.pdf]

Supplementary Videos

Video S1 *In-situ* TEM observation of nanoscopic deformation of nt-Cu nanopillar under compression at a relatively high strain rate of about 3.1×10^{-4}/s (10 times speed up).

Video S2 *In-situ* TEM observation of nanoscopic deformation of nt-Cu nanopillar under compression at a medium strain rate of about 1.3×10^{-4}/s (10 times speed up).

Videos S3-S6 *In-situ* TEM observation of nanoscopic deformation of nt-Cu nanopillars under compression at an ultralow strain rate of 2.3×10^{-5}/s (5 times speed up).
